# Condensation of Plasmid DNA Enhances Mitochondrial Association in Skeletal Muscle Following Hydrodynamic Limb Vein Injection

**DOI:** 10.3390/ph7080881

**Published:** 2014-08-21

**Authors:** Yukari Yasuzaki, Yuma Yamada, Yutaka Fukuda, Hideyoshi Harashima

**Affiliations:** Laboratory for molecular design of pharmaceutics, Faculty of Pharmaceutical Sciences, Hokkaido University, Kita-12, Nishi-6, Kita-ku, Sapporo 060-0812, Japan; E-Mails: yukari-y@mail.sci.hokudai.ac.jp (Y.Y.); u-ma@pharm.hokudai.ac.jp (Y.Y.); yutaka_f@ec.hokudai.ac.jp (Y.F.)

**Keywords:** mitochondrial gene delivery, hydrodynamic limb vein injection, condensed pDNA, mitochondrial gene therapy

## Abstract

Mitochondrial gene therapy and diagnosis have the potential to provide substantial medical benefits. However, the utility of this approach has not yet been realized because the technology available for mitochondrial gene delivery continues to be a bottleneck. We previously reported on mitochondrial gene delivery in skeletal muscle using hydrodynamic limb vein (HLV) injection. HLV injection, a useful method for nuclear transgene expression, involves the rapid injection of a large volume of naked plasmid DNA (pDNA). Moreover, the use of a condensed form of pDNA enhances the nuclear transgene expression by the HLV injection. The purpose of this study was to compare naked pDNA and condensed pDNA for mitochondrial association in skeletal muscle, when used in conjunction with HLV injection. PCR analysis showed that the use of condensed pDNA rather than naked pDNA resulted in a more effective mitochondrial association with pDNA, suggesting that the physicochemical state of pDNA plays a key role. Moreover, no mitochondrial toxicities in skeletal muscle following the HLV injection of condensed pDNA were confirmed, as evidenced by cytochrome c oxidase activity and mitochondrial membrane potential. These findings have the potential to contribute to the development for *in vivo* mitochondrial gene delivery system.

## 1. Introduction

Mutations and defects in the mitochondrial genome form the basis of a variety of human diseases, many of which involve mitochondrial dysfunctions [1,2,3,4]. Therefore, mitochondrial gene therapy and diagnosis could be expected to offer substantial medical benefits, however, the utility of this strategy has not yet been realized because the technology for *in vivo* mitochondrial gene delivery is a bottleneck. We previously reported on the potential for using hydrodynamic limb vein (HLV) injection for achieving mitochondrial gene delivery targeted to mammalian skeletal muscle tissue [5]. Skeletal muscle represents an attractive target tissue for mitochondrial gene therapy, because mitochondrial genomic dysfunctions in skeletal muscle are largely associated with various mitochondrial diseases [2,4]. The HLV injection procedure, a useful method for nuclear transgene expression in skeletal muscle, involves the rapid injection of a large volume of naked plasmid DNA (pDNA) into the distal vein of a limb [6,7,8,9]. Using PCR analysis, we demonstrated the HLV injection technique could be used to deliver naked pDNA into myofibrillar mitochondria and that it had no influence on mitochondrial function [5].

**Figure 1:**
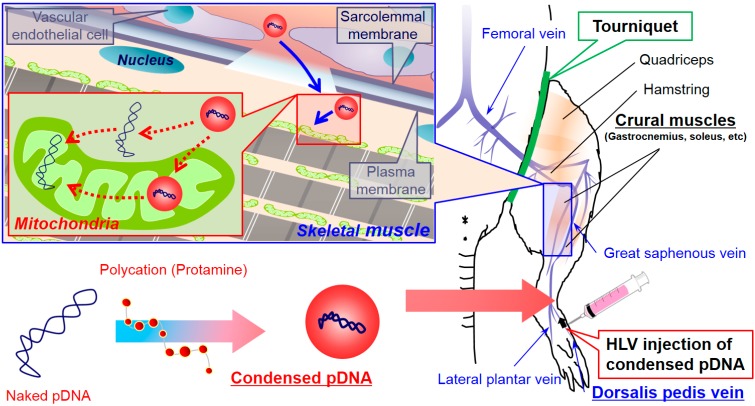
Schematic image of mitochondrial gene delivery in skeletal muscle by HLV injection of condensed pDNA. pDNA was condensed with a polycation (Protamine) and the condensed pDNA was intravenously injected into the dorsalis pedis vein via HLV injection, which is similar to a previously reported method [5]. It is generally thought that the condensed pDNA is internalized into cells via hydrodynamic force, and mitochondrial localization of pDNA may be achieved.

In this study, we focused on the condensation of pDNA to enhance mitochondrial association, since it has been reported that, when condensed pDNA is used in HLV injection, it is even more effective than naked pDNA in achieving nuclear transgene expression in skeletal muscle [10,11,12]. Figure 1 shows the schematic image of mitochondrial gene delivery in skeletal muscle by HLV injection of condensed pDNA. A sufficient volume of saline is used to facilitate extravasation of the condensed pDNA from the vasculature and into the muscle tissue through multiple physical barriers. Hydrodynamic force could induce the transient opening cellular membrane to permit the condensed pDNA to be internalized into cells. Finally, the localization of pDNA in mitochondria may also be achieved by alternate, currently unknown mechanisms.

The objective of this study was to compare naked pDNA and condensed pDNA for mitochondrial association, in conjunction with HLV injection. We first determined the optimal conditions for condensing pDNA by measuring the sizes and zeta-potentials of the particles. We then, using PCR analysis, investigated the effects of injection volume and the condensation of pDNA on mitochondrial association by HLV injection. Finally, we assessed mitochondrial toxicity in skeletal muscle following the HLV injection of condensed pDNA, in terms of cytochrome c oxidase (COX) activity and mitochondrial membrane potential.

## 2. Experimental Section

### 2.1. Materials

The pcDNA3.1 (+)-luc plasmid was constructed by inserting the firefly luciferase gene (Hind III-Xba I fragment) of the pGL3-Control plasmid (Promega, Madison, WI, USA) into the pcDNA3.1 (+) plasmid (Life Technologies Corporation, Carlsbad, CA, USA) pretreated with the same restriction enzymes. The pDNA was purified using an Endfree Plasmid Giga Kit (Qiagen GmbH, Hilden, Germany). Oligonucleotides were purchased from Sigma Genosys Japan (Ishikari, Japan) in purified form. Protamine, the native protamine sulfate from salmon milt, was purchased from Calbiochem (Darmstadt, Germany). Tetramethylrhodamine (TMRM) and MitoTracker Deep Red 633 (MTDR) were purchased from Life Technologies Corporation. All other chemicals used were commercially available, reagent-grade products.

### 2.2. Experimental Animals

Female Wistar Hannover rats (7–9 weeks old) were purchased from Sankyo Labo Service (Sapporo, Japan). Rats with body weight in the range 150–180 g were used in all experiments. All animal protocols were approved by the institutional animal care and research advisory committee at the Faculty of Pharmaceutical Sciences, Hokkaido University, Sapporo, Japan (date: 22 March 2013, registration No. 13-0062).

### 2.3. Preparation of Condensed pDNA Particles

To prepare condensed pDNA particles, solutions of pDNA (0.3 mg/mL) and protamine were mixed in 10 mM HEPES buffer (pH 7.4) under vortexing at various nitrogen/phosphate (N/P) ratios. Particle diameters were measured using a quasi-elastic light scattering method by a Zetasizer Nano ZS (Malvern Instruments, Worcestershire, UK). The zeta-potentials, the nature of the electrostatic potential near the surface of a particle, were determined by laser doppler micro-electrophoresis in which the velocity of the particles in an electric field were measured using the Zetasizer Nano ZS.

### 2.4. Validation of pDNA Condensation and Decondensation

To evaluate the release of pDNA, condensed pDNA with protamine at an N/P ratio of 2.3 was subjected to agarose gel electrophoresis before and after treatment with a polyanion. Prior to electrophoresis, the samples were treated with a polyanion solution, *i.e.*, 1 mg/mL poly(l-aspartic acid) (pAsp), for 20 min at 25 °C to release the pDNA. A 0.1-μg sample of pDNA was subjected to electrophoresis. Electrophoresis was performed on a 1% agarose gel in TAE (40 mM Tris-HCl, 40 mM acetic acid, 1 mM EDTA, pH 8.0) at 100 V for 20 min. The gel was stained with ethidium bromide.

### 2.5. Hydrodynamic Injection into the Limb Vein of Rats

Rats were anesthetized with a pentobarbital (37.5 mg/kg) solution via an intraperitoneal injection. Prior to each pDNA injection, a tourniquet was placed on the upper hind limb to restrict blood flow into and out of the hind limb. Basically, naked pDNA or condensed pDNA suspensions (3 mL) containing 184 μg pDNA were injected in 20 s from a distal site of the dorsalis pedis vein. At 2 min after the injection, the tourniquet was released. A schematic diagram of this methodology, which was previously reported for the HLV injection of naked pDNA [5], is shown in Figure 1.

### 2.6. Quantification of pDNA in Mitochondria-Enriched Fraction after HLV Injection

The experimental protocol was designed based on findings obtained in our previous report [5]. At 24 h postinjection, the rats were sacrificed, the crural muscles harvested, and the mitochondria-enriched fraction was then obtained from the tissue (see the Supplemental Material for details). We first extracted total DNA including mtDNA from muscle tissue using a GenElute Mammalian Genomic DNA Miniprep Kit (Sigma-Aldrich Co., St Louis, MO, USA) to determine the copy numbers of mtDNA per mg of muscle protein (mtDNA_muscle_ [mtDNA-copy/mg muscle protein]). Copy numbers of mtDNA were estimated by quantitative real-time PCR (q-PCR) (see the Supplemental Material for details), and concentrations of the muscle protein were measured using a BCA protein assay kit (Pierce, Rockford, IL, USA). We next extracted pDNA and mtDNA from the mitochondria-enriched fraction using SepaGene (Sanko Jun-yaku, Tokyo, Japan), and the copy numbers of pDNA and mtDNA were measured by q-PCR to determine the amount of pDNA per mtDNA in mitochondria-enriched fraction (pDNA_mt_ [fg pDNA/mtDNA-copy]). The amount of pDNA in the mitochondria-enriched fraction of muscle tissues were calculated as follows:

Mitochondrial association with pDNA [fg pDNA/mg muscle protein] = mtDNA_muscle_ [mtDNA-copy/mg muscle protein] × pDNA_mt_ [fg pDNA/mtDNA-copy].

### 2.7. DNase Treatment of Mitochondria-Enriched Fraction Following HLV Injection of pDNA

The experimental protocol was designed based on findings obtained in our previous report [5]. At 24 h after the HLV injection of condensed pDNA suspensions (184 μg, 3 mL), the rats were sacrificed, the crural muscles were harvested, and a mitochondria-enriched fraction was then obtained from the tissue. The mitochondria-enriched fraction in a total volume of 38 μL was incubated with 20 U of Recombinant DNase I (RNase-free) (Takara Bio Iic., Shiga, Japan) at 37 °C for 30 min, and 2 μL of 0.5 M EDTA (pH 8.0) was then added and the suspension was incubated at 80 °C for 2 min to stop the reaction. A similar procedure was carried out, but without the DNase I treatment to produce a sample that had not been subjected to DNase I treatment. We next extracted pDNA and mtDNA from the mitochondria-enriched fraction, and the amount of pDNA and mtDNA were measured by q-PCR. Finally we determined the amount of pDNA per mtDNA in mitochondria-enriched fraction (ng pDNA/mg mtDNA).

### 2.8. COX Staining

The experimental protocol was designed based on findings obtained in our previous report [5]. At 24 h postinjection, the rats were sacrificed, the crural muscles containing the gastrocnemius/soleus were harvested, and COX staining was performed on the dried tissue sections. COX is the collective name for the part of the oxidative respiratory chain of enzymes that are located exclusively in the mitochondria of cells. Briefly, 5-μm thick sections of tissues were prepared on coverslips with a Tissue-Tek Cryo 3D cryostat (Skura Finetek Japan Co., Ltd, Tokyo, Japan). The sections were then placed in the incubating medium (0.1 M CH_3_COONa, 2 mg/mL di-amino benzidinetetrachloride (DAB), 0.1% MnCl_2_, 0.1% H_2_O_2_, pH 5.5) for 1 h at 37 °C. The section was washed with deionized distilled H_2_O and then treated with 1% CuSO_4_ for 5 min. After washing the section with deionized distilled H_2_O, it was dehydrated in an ascending series of alcohols (50%, 70%, 80%, 90%, 95%, 100% × 2) and cleared in xylene. The section was mounted and then observed by microscopy. The use of DAB results in a brown insoluble compound at the site of cytochrome oxidase activity. In this experiment, we used COX-positive cells, which were stained brown, indicating that they had COX activity. We also calculated the ratios of COX-positive cells and the values are indicated on each image.

### 2.9. Observations of Mitochondrial Membrane Potentials in Muscle Tissue

The experimental protocol was designed based on findings obtained in our previous report [5]. At 24 h postinjection, the rats were sacrificed and the crural muscles containing the gastrocnemius/soleus were harvested. The muscle tissues were treated with TMRM and MTDR to stain mitochondria, and were then observed by confocal laser scanning microscopy (CLSM). TMRM and MTDR were purchased from Life Technologies Corporation. TMRM, a conventional fluorescent stain for mitochondria, is easily washed out once the mitochondria experience a loss in membrane potential. On the other hand, MTDR is also selective for mitochondria and the stain is retained, even when mitochondrial membrane potential is lost. This experiment allowed us to evaluate the extent to which the hydrodynamic injection affected mitochondrial membrane potential. Briefly, the muscle tissues were incubated for 20–30 min in Hank's buffered salt solution containing TMRM (final concentration, 1 μM), MTDR (final concentration, 1 μM). Fluorescent images were obtained by CLSM (Nikon A1; Nikon Co. Ltd., Tokyo, Japan). The tissue specimens were excited at a wavelength of 561 nm by a DPSS laser. A series of images were obtained using a Nikon A1 confocal imaging system equipped with a water immersion objective lens (Plan Apo 60_1.20 PFS WI) and a 1st dichroic mirror (405/488/561/640). The two fluorescence detection channels (Ch) were set to the following filters: Ch1: 595/50 (red color) for TMRM and Ch2: 700/75 (cyan pseudo color) for MTDR. When the mitochondrial membrane potential was depolarized, the muscle tissues were treated with carbonyl cyanide 4-(trifluoromethoxy)phenylhydrazone (FCCP, Sigma), a mitochondrial uncoupler, before observation (final concentration of FCCP, 100 μM).

### 2.10. Statistical Analysis

**Figure 2:**
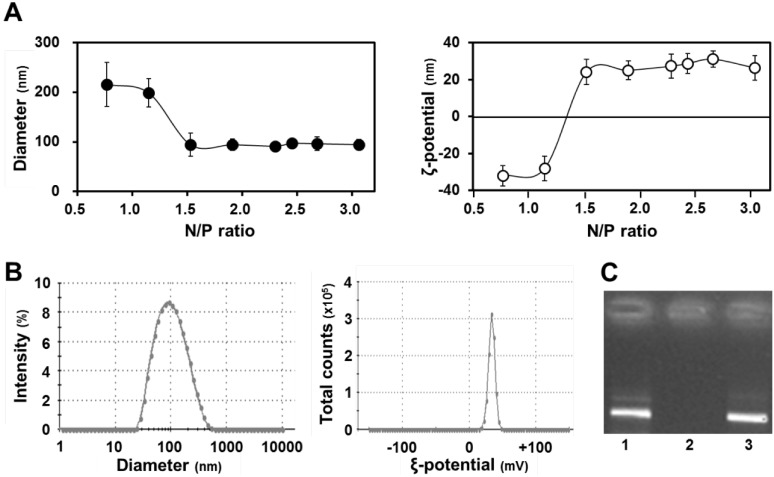
Characteristics of condensed pDNA. (**A**) Diameters (left) and ξ potentials (right) of condensed pDNA prepared using protamine at a series of N/P ratios. Data represent the mean ± S.D. (*n* = 4); (**B**) Distribution of diameter (left) and ξ potential (right) of condensed pDNA with protamine at an N/P ratio of 2.3; (**C**) Gel electrophoresis data for the release of pDNA from condensed pDNA. Naked pDNA (lane 1) and pDNA condensed with protamine were subjected to agarose gel electrophoresis before (lane 2) and after (lane 3) pAsp treatment.

**Figure 3:**
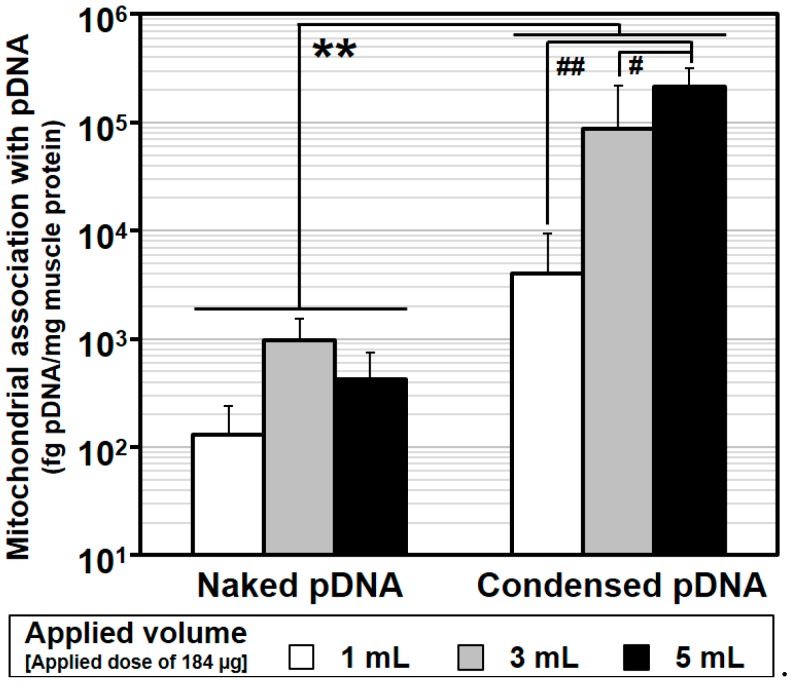
Comparison between naked pDNA and condensed pDNA for mitochondrial association with pDNA as the result of HLV injection. After the HLV injection of naked pDNA or condensed pDNA particles, the crural muscles were harvested, the pDNA in the mitochondria-enriched fraction was measured using q-PCR. Regarding the values for naked pDNA, these data were previously reported [5]. Open columns represent 184 μg/1 mL, gray columns represent 184 μg/3 mL and closed columns represent 184 μg/5 mL. Bars indicate the mean ± S.D. (*n* = 3–5). ** Significant differences of mitochondrial association with pDNA between naked pDNA and condensed pDNA (*p* < 0.001 by two-way ANOVA, followed by Bonferroni correction). Significant differences (## *p* < 0.01, # *p* < 0.05) of mitochondrial association with pDNA among injection volumes for naked pDNA and condensed pDNA by two-way ANOVA, followed by Bonferroni correction.

**Figure 4:**
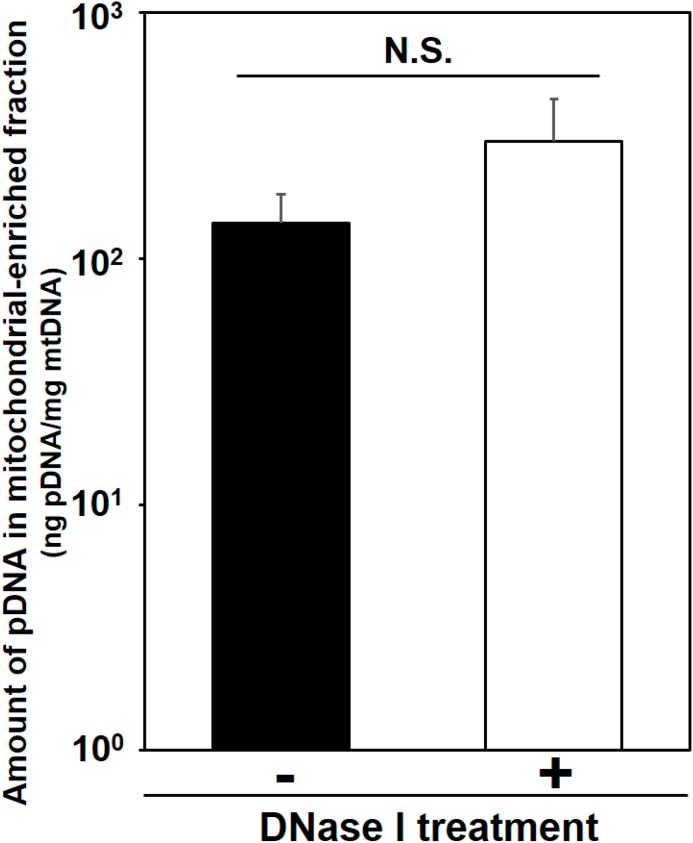
Detection of exogenous pDNA in the mitochondria-enriched fraction before and after DNase treatment. At 24 h after HLV injection of the condensed pDNA, the crural muscles were harvested, and the pDNA in mitochondria-enriched fraction was then measured using q-PCR, before and after treatment with DNase I. Bars indicate means ± S.D. (*n* = 3). Statistical analysis was performed by a two-tailed unpaired Student’s *t*-test. N.S. indicates non-significant difference.

**Figure 5:**
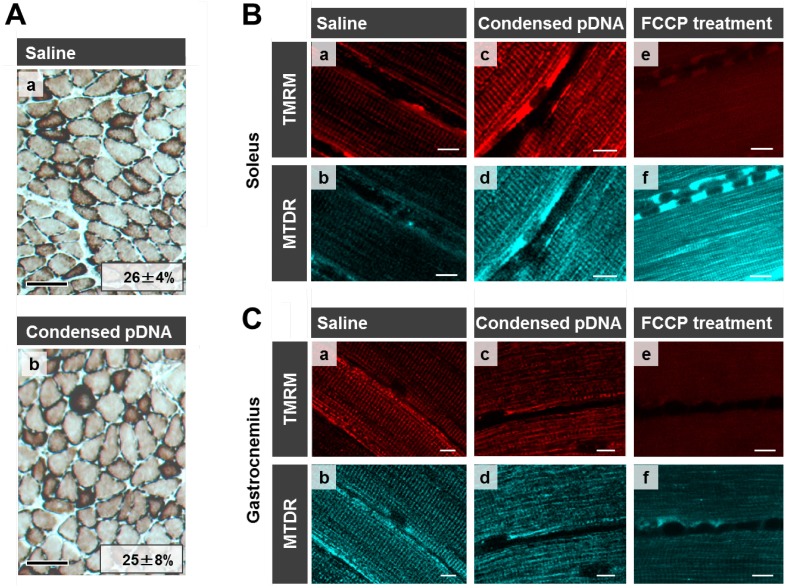
Evaluation of mitochondrial toxicity following HLV injection. (**A)** COX staining of skeletal muscle following HLV injection. Frozen cross-sections (10 μm thickness) of HLV injection performed skeletal muscle were prepared, followed by COX staining. The section was then observed by microscopy; saline administered muscle (**a**) and condensed pDNA administered muscle (**b**). Scale bars, 100 μm. In this experiment, cells were stained brown when the cells have the cytochrome oxidase activity (COX-positive cells). We also calculated the ratios of COX-positive cells and the values for each image as indicated. Data represent the mean ± S.D. (*n* = 3). Statistical analysis was performed by a two-tailed unpaired Student’s t-test (*p* = 0.95). (**B**,**C)** Evaluation of mitochondrial membrane potential in skeletal muscles following HLV injection. At 24 h post HLV injections, the soleus (**B**) or gastrocnemius (**C**) of crural muscles were harvested, and mitochondria were then stained with TMRM (red color; a,c,e) and MTDR (cyan pseudo color; b,d,f). The staining of mitochondria with TMRM is dependent on the membrane potential, while MTDR can stain mitochondria even when membrane potential is lost. The muscle tissues were observed using CLSM. Scale bars, 10 μm.

The diameters and zeta-potentials of condensed pDNA formed using protamine at a series of N/P ratios were measured respectively. Each value shown in Figure 2A is represented by the mean ± S.D (*n* = 4). Mitochondrial association with pDNA compared between naked pDNA and condensed pDNA at various injection volumes were evaluated. Each value shown in Figure 3 is represented by the mean ± S.D. (*n* = 3–5). Statistical significances between naked pDNA and condensed pDNA, and among injection volumes were examined by the two-way ANOVA, followed by Bonferroni correction. Detection of exogenous pDNA in the mitochondria-enriched fraction before and after DNase treatment were evaluated. Each value shown in Figure 4 is represented by the mean ± S.D. (*n* = 3). Statistical significances between before and after treatment with DNase I were examined by the two-tail unpaired student’s t-test. The ratios of COX-positive cells compared between saline administered muscle and condensed pDNA administered muscle were evaluated. Each value shown in Figure 5A is represented by the mean ± S.D. (*n* = 3). Statistical significances between saline and condensed pDNA administered muscles were examined by the two-tail unpaired student’s *t*-test. Levels of *p* < 0.05 were considered to be significant.

## 3. Results and Discussion

### 3.1. Condensation of pDNA and the Evaluation of the Physiochemical Properties

The optimal conditions were determined for condensing pDNA with protamine, a condenser showing efficient DNA release [13,14]. pDNA was mixed with protamine at several N/P ratios to form nanoparticles and their diameters and ζ-potentials were then measured (Figure 2A). Figure 2A (left panel) shows the diameters of the condensed pDNA, in which particles with diameters of ~100 nm were formed at N/P ratios higher than 1.5. Figure 2A (right panel) shows the ζ potentials of condensed pDNA, where the charges of particles had changed from minus to plus when the N/P ratio was increased. In this experiment, we used small positively charged particles that were formed at an N/P ratio of 2.3. As shown in Figure 2B, the condensed pDNA was a positively charged nanoparticle with a homogeneous structure. A dynamic light scattering analysis indicated a single population that was small in size (left panel, Figure 2B). Furthermore, the ζ potential for the carrier also exhibited a single peak (right panel, Figure 2B).

We also evaluated the stability of the condensed pDNA with protamine by agarose gel electrophoresis before and after treatment with a polyanion, which inhibits the development of electrostatic interactions between pDNA and the cationic protamine. (Figure 2C). In this experiment, we observed the fluorescent band of pDNA, when pDNA is released from the condensed pDNA. Gel electrophoresis data showed that the pDNA was condensed with protamine under normal conditions (lane 2, Figure 2C). On the other hand, pDNA was easily released from the condensed pDNA by protamine in the presence of a counter polyanion such as poly(l-aspartic acid) (pAsp) (lane 3, Figure 2C). We hypothesized that pDNA would be released from the condensed pDNA during the period that the q-PCR experiment was performed, because a buffer containing a polyanion was used during the q-PCR experiment. Thus, we concluded that it is possible to compare mitochondrial DNA association between naked pDNA and condensed pDNA, when the pDNA was condensed with protamine.

### 3.2. Comparison of Mitochondrial Association with pDNA by HLV Injection between Naked pDNA and Condensed pDNA

The use of naked pDNA and condensed pDNA in mitochondrial association were compared, after the HLV injection of 184 μg of pDNA in 1–5 mL of solution (Figure 3). In this experiment, pDNA was intravenously injected within a period of 20 s into the distal hind limb of rats, as previously reported [5]. At 24 h postinjection, the rats were sacrificed, and the pDNA in the mitochondria-enriched fraction of crural muscles was quantified using q-PCR.

Figure 3 shows a comparison of mitochondrial association with pDNA by HLV injection between naked pDNA and condensed pDNA and the statistical analysis by a two-way ANOVA. Regarding the values for naked pDNA, previous data were used [5]. Based on the results, the mitochondrial association with condensed pDNA was significantly higher than that of naked pDNA for any injection volume (**Significant difference (*p* < 0.001), Figure 3). It is noteworthy that the mitochondrial association with condensed pDNA was ~1000 fold higher than that of naked pDNA in the case of a 5 mL injection volume (black column, Figure 3). We also compared the mitochondrial DNA association as a function of injection volume for naked pDNA and condensed pDNA, respectively. In the case of naked pDNA, the values were comparable. On the other hand, in the case of condensed pDNA, the value for a 5 mL injection volume (black column in right, Figure 3) was significantly higher than the others. These results suggest that, to achieve the efficient mitochondrial association with condensed pDNA by HLV injection, a high injection volume might be required.

As shown in Figure 3, the use of condensed pDNA increased the amount of pDNA in the mitochondria-enriched fraction following HLV injection compared to naked pDNA. Here, we considered the reasons for why condensation of pDNA enhanced mitochondrial association following HLV injection. In previous reports regarding nuclear transgene expression using mice and HLV injections, Itaka *et al.* showed that condensed pDNA resulted in about a 200-fold increase in the amount of intact pDNA in muscle compared to naked pDNA [10]. This report prompted us to consider that condensed pDNA, which has a small rigid structure, may have easy access to myofibrillar mitochondria, which are present on muscle fibers far from blood vessels. On the other hand, the results of previous *in vitro* experiments suggest that the mitochondrial import of large-sized cargoes might be inhibited in many cytoskeletons and a high density of cell components inside the cells [15]. Hydrodynamic force may assist condensed pDNA in accessing mitochondria, even in such a cell environment, although the mitochondrial import of condensed pDNA would be hindered by intracellular barriers. It is also presumed that condensed pDNA with a positive charge would bind readily to mitochondria, because a high negative potential would be maintained.

Another possibility is that the use of pDNA condensed with polycations could influence the levels of exogenous pDNA in myofibrillar mitochondria. Previous reports showed that the amounts of naked pDNA in the nuclei of liver cells decreased to 1/10 at 24 h after hydrodynamic injection into tail vein of mice [16]. Thus, the more than a 100–1000 fold increase in the levels of exogenous pDNA in the mitochondria-enriched fraction (Figure 3) cannot be explained only by protecting pDNA from degradation, although the target organ and organelle were different between the current study and the previous study. Based on previous reports and our results, we concluded that the high levels of exogenous pDNA in the mitochondria-enriched fraction (when condensed pDNA was used) can be explained, not only by the protection of pDNA from degradation but also by an enhancement in the mitochondrial association with pDNA.

### 3.3. DNase Treatment of Mitochondria-Enriched Fraction Following HLV Injection of Condensed pDNA

In the previous study, we showed that mitochondrial delivery of naked pDNA by HLV injection achieved the localization of exogenous pDNA in inside a mitochondrion, based on the DNase I digestion experiment [5]. As shown in Figure 4, we detected exogenous pDNA in the mitochondria-enriched fraction that had been treated with DNase I, after the HLV injection of condensed pDNA. As a result, the levels of exogenous pDNA in the mitochondria-enriched fraction following the HLV injection of condensed pDNA (184 μg, 3 mL) were comparable to the values before and after the DNase I treatment (Figure 4). We also confirmed that pDNA was released from the condensed pDNA when mixed with the mitochondria-enriched fraction, and DNase I digested the pDNA (data not shown). Our results suggest the possibility that the HLV injection of condensed pDNA resulted in the localization of exogenous pDNA inside mitochondria, although the mechanism responsible for this remains unknown. This issue will be investigated in the future.

### 3.4. Evaluation of Mitochondrial Toxicity in Skeletal Muscle after HLV Injection of Condensed pDNA

Since the use of HLV injection for the efficient delivery of pDNA involves rather severe conditions, a toxicity assessment is an important issue that is related to the potential therapeutic utility of this methodology. Therefore, we previously investigated mitochondrial toxicity following HLV injection, and an evaluation of COX activity and mitochondrial membrane potentials showed that the HLV injection had no significant effect on mitochondrial function [5].

Here we evaluated mitochondrial toxicity after the HLV injection of condensed pDNA, which resulted in a more efficient mitochondrial association with pDNA. Myofibrillar mitochondrial activity was evaluated by COX staining, after performing an HLV injection (Figure 5A). Saline administered muscle was used as a positive control for COX staining where COX-positive cells are stained brown [Figure 5A(a)]. COX-positive cells were observed in skeletal muscles after the HLV injection of condensed pDNA [Figure 5A(b)]. It was also confirmed that the ratios of COX-positive cells between saline administered muscle (26% ± 4%) and condensed pDNA administered muscle (26% ± 7%) were comparable. The results indicate that the mitochondria maintained COX activity in skeletal muscles after the HLV injection of condensed pDNA.

Mitochondrial membrane potentials of the soleus of crural muscle following HLV injection were also evaluated (Figure 5B). The staining of mitochondria with tetramethylrhodamine (TMRM) (red color; a,c,e, Figure 5B) is dependent on the membrane potential, while MitoTracker Deep Red 633 (MTDR) stains mitochondria (cyan pseudo color; b,d,f, Figure 5B), even when the membrane potential is lost. In the case of saline administered muscle (a,b) and condensed pDNA administered muscle (c,d), the mitochondria were extensively stained with TMRM at comparable levels, indicating that most of the mitochondria in skeletal muscles maintained their membrane potential.

We also confirmed that mitochondria were incompletely stained by TMRM, when the mitochondrial membrane potential was depolarized, in the case where muscles were treated with FCCP (a mitochondrial uncoupler) (e,f in Figure 5B). These results indicate that the HLV injection of condensed pDNA does not cause a significant decrease in the mitochondrial membrane potential, compared to the FCCP treatment. A similar tendency regarding mitochondrial membrane potentials was observed in the case of the gastrocnemius of crural muscles (Figure 5C).

## 4. Conclusions

Condensation of pDNA enhances the mitochondrial association with pDNA following HLV injection. The results suggest that the physicochemical state of pDNA appears to play key role in mitochondrial delivery by HLV injection. Moreover, the evaluation of COX activity and mitochondrial membrane potentials showed that HLV injection was not toxic to mitochondria. These findings can contribute to the development of *in vivo* mitochondrial gene delivery systems. Planned future studies involve attempts to achieve *in vivo* mitochondrial transgene expression by HLV injection in conjunction with experts in mitochondrial molecular biology. Studies directed toward this goal are currently in progress.

## Supplementary Files

Supplementary File 1
