# Supplementary material for: Condensation of Plasmid DNA Enhances Mitochondrial Association in Skeletal Muscle Following Hydrodynamic Limb Vein Injection
Source: Pharmaceuticals (Basel). 2014 Aug 21;7(8):881–93. doi: 10.3390/ph7080881 (PMC4167204; doi:10.3390/ph7080881)
Supplement: Supplementary File 1 [file pharmaceuticals-07-00881-s001.docx]

**Supplemental Materials**

Materials and Methods

Isolation of Mitochondria from Rat Skeletal Muscle

A mitochondria-enriched fraction was isolated from a homogenate by differential centrifugation, as described in a previous report [1,2]. At 24 h postinjection, the rats were sacrificed and the crural muscles were harvested and minced with scissors, and the minced tissue was added to 1 mL of ice-cold mitochondrial isolation buffer [MIB: 250mM sucrose, 2 mMTris–HCl, 1 mM EDTA, pH 7.4]. The suspension was homogenized using a PreCellys, and centrifuged at 800 *g* for 5 min at 4 °C. A 500 μL of supernatant was transferred into ice-cold tubes containing 500 μL of MIB, and then centrifuged at 800 *g* for 5 min at 4 °C. A 500 μL aliquot of the supernatant was transferred into ice-cold tubes containing 500 μL of MIB, and the suspensions were centrifuged at 7,500 *g* for 10 min at 4 °C.
The pellets were washed with EDTA-free MIB, and the pellets were resuspended in EDTA-free MIB and the resulting suspensions were used as the mitochondria-enriched fraction. The purity of the mitochondria-enriched fraction was confirmed by western blotting to detect organelle specific protein [3].

Quantification of DNA by q-PCR

q-PCR was performed using a 7500 Real Time PCR system (Applied Biosystems, Foster City, CA) with the following parameters: an initial denaturation at 95 °C for 10 min, followed by 40 cycles at 95 °C for 15 s and 60 °C for 1 min. SYBR Green qPCR Mastermix (TOYOBO, Osaka, Japan) was used to indicate target gene amplification and the specificity of amplification was examined by automated dissociation stage analysis. A portion of the extracted DNA was analyzed for pDNA or mtDNA, using primers Luc (+) and Luc (−) and primers Rat D-loop (+) and Rat D-loop (−) [Table S1]. The copy number of pDNA or mtDNA was absolutely quantified using real time PCR. To quantify the mtDNA, a standard curve was prepared using mtDNA obtained from rat liver mitochondria. Rat liver mitochondria was prepared as described in a previous report [1,2], and mtDNA was isolated from the rat liver mitochondria by alkaline extraction, as described in a previous report [4].

**Table S1.** Primers used for the PCR to quantify pDNA and mtDNA.

| **Primers** | **Nucleotide sequence** | **Purpose** |
| --- | --- | --- |
| Luc (+) | 5'-GGTCCTATGATTATGTCCGGTTATG-3' | Quantification for pDNA (exogenous gene) |
| Luc (−) | 5'-ATGTAGCCATCCATCCTTGTCAAT-3' |  |
| Rat D-loop (+) | 5'-GCTTCTTCGCATTCCACTTC-3' | Quantification for mtDNA (exogenous gene) |
| Rat D-loop (−) | 5'-TCGGAGTTTAATCCTGTGGG-3' |  |

Oligonucleotides were purchased from Sigma Genosys Japan (Ishikari, Japan) in purified form.

**References**

1. Shinohara, Y.; Almofti, M.R.; Yamamoto, T.; Ishida, T.; Kita, F.; Kanzaki, H.; Ohnishi, M.; Yamashita, K.; Shimizu, S.; Terada, H. Permeability transition-independent release of mitochondrial cytochrome c induced by valinomycin. *Eur. J. Biochem.* **2002**, *269*, 5224–5230.
2. Yamada, Y.; Akita, H.; Kamiya, H.; Kogure, K.; Yamamoto, T.; Shinohara, Y.; Yamashita, K.; Kobayashi, H.; Kikuchi, H.; Harashima, H. MITO-Porter: A liposome-based carrier system for delivery of macromolecules into mitochondria via membrane fusion. *Biochim. Biophys. Acta* **2008**, *1778*, 423–432.
3. Yasuzaki, Y.; Yamada, Y.; Kanefuji, T.; Harashima, H. Localization of exogenous DNA to mitochondria in skeletal muscle following hydrodynamic limb vein injection. *J. Control. Release* **2013**, *172*, 805–811.
4. Palva, T.K.; Palva, E.T. Rapid isolation of animal mitochondrial DNA by alkaline extraction. *FEBS Lett.* **1985**, 192, 267–270.

© 2014 by the authors; licensee MDPI, Basel, Switzerland. This article is an open access article distributed under the terms and conditions of the Creative Commons Attribution license (http://creativecommons.org/licenses/by/3.0/).
